# Tinct. Iron in Nasal Polypus

**Published:** 1875-08

**Authors:** 


Tincture of Chloride of Iron for Nasal Polypus.—Dr. G. Troup Maxwell (*Phil. Med. Times*) injects into the nostril one drachm tincture of iron, diluted with one drachm water, holding the head back so that the liquid shall come in contact with the polypus. This he repeats daily, with the effect of producing a complete cure in from four to seven or eight days. It causes but slight pain, and does not prevent the patient from attending to his business.

The Transfusion of Goat's Blood into an Irishman, according to an Eastern newspaper, had a most extraordinary effect. He went to butting, and knocked down everybody in his reach. When it was supposed the effects had entirely gone off, the man went to church, and whilst there a corpuscle of the goat's blood found its way into his brain, causing him to butt the priest with dangerous violence.

